# Circular RNA as a Novel Biomarker for Diagnosis and Prognosis and Potential Therapeutic Targets in Multiple Myeloma

**DOI:** 10.3390/cancers14071700

**Published:** 2022-03-27

**Authors:** Alessandro Allegra, Nicola Cicero, Alessandro Tonacci, Caterina Musolino, Sebastiano Gangemi

**Affiliations:** 1 Department of Human Pathology in Adulthood and Childhood “Gaetano Barresi”, Division of Hematology, University of Messina, 98125 Messina, Italy; cmusolino@unime.it; 2 Department of Biomedical and Dental Sciences and Morphofunctional Imaging, University of Messina, 98125 Messina, Italy; nicola.cicero@unime.it; 3 Clinical Physiology Institute, National Research Council of Italy (IFC-CNR), 56124 Pisa, Italy; atonacci@ifc.cnr.it; 4 Allergy and Clinical Immunology Unit, Department of Clinical and Experimental Medicine, University of Messina, 98125 Messina, Italy; gangemis@unime.it

**Keywords:** long non-coding RNA, circular RNA, multiple myeloma, chemoresistance, prognosis, bortezomib, microRNA, gene expression, epigenetics

## Abstract

**Simple Summary:**

Circular (circ)RNAs are closed RNAs able to influence a wide range of biological systems at least in part by interacting with microRNAs. CircRNAs expressed in the hematopoietic compartment have been progressively identified as modulators of the pathological features of hematological cells. In particular, several circRNAs were found to enhance or inhibit tumor progression in blood malignancies such as multiple myeloma. We discuss the usefulness of circRNAs as diagnostic and prognostic markers and their potential value as therapeutic targets in multiple myeloma patients.

**Abstract:**

Circular RNAs (circRNAs) are a novel type of covalently closed RNAs involved in several physiological and pathological processes. They display tissue-specific expression and are constant, abundant, and highly conserved, making them perfect markers for diagnosis and prognosis. Several studies have proposed that circRNAs are also differentially produced in malignancies where they have oncogenic effects. Furthermore, circRNAs affecting microRNAs modify the expression profile of several transcription factors which play essential roles in tumors. CircRNAs within the hematopoietic compartment were identified as modulators of mechanisms able to enhance or suppress tumor progression in blood malignancies. Moreover, several circRNAs were suggested to confer resistance to the conventional drugs employed in hematopoietic cancers. In this review, we highlight the growing role and the controlling mechanisms by which circRNAs modify multiple myeloma genesis. We propose that circRNAs can be considered as potential diagnostic and prognostic markers, can induce chemoresistance, and might represent novel therapeutic targets for multiple myeloma.

## 1. Introduction

### General Considerations on circRNAs

In the human genome, only about 2% of the genes can be transformed into proteins, while the remainder are represented by non-coding RNAs (ncRNAs), which are able to control cellular functions including growth, differentiation and programmed cell death [1]. They are classified into short and long ncRNAs, corresponding to their extent, and circular RNAs (circRNAs).

Short ncRNAs, such as microRNAs (miRNAs), and piwi-interacting RNAs, are ncRNAs 19–25 nucleotides long which control gene expression that affects the target mRNAs or suppresses translation [2,3,4]. Long ncRNAs (lncRNAs) can be detected by longer sequences and are broadly present in eukaryotic cells, with from about 5400 to more than 10,000 lncRNA transcripts in the human genome [5]. Finally, circRNAs are RNA molecules covalently closed without 5′ or 3′ ends and a poly-A tail and have fundamental effects in physiology and in several pathologic conditions. They were originally discovered in an RNA virus in 1976 and afterwards in eukaryotes, with a number of the 413,657 types having been described by 2020 [6]. 

According to their structure, circRNAs can be divided into several groups, such as exonic circRNAs (ecircRNAs) made by exons, circular intronic RNAs (ciRNAs) made by introns, and exon-intron circRNAs (EIciRNAs) made by both exons and introns [7]. In fact, the majority of circRNAs include exons from genes that code proteins, but they also stem from introns, untranslated regions, intergenic regions, and antisense transcripts of genes. Furthermore, other forms of circRNAs have been identified, including fusion circRNA (f-circRNAs), read-through circRNAs (rt-circRNAs), and mitochondria-encoded circRNA (mecciRNAs) [8,9]. 

These different kinds of circRNA are located in various cell compartments and, although circRNAs are produced in the nucleus, a large amount is generally located in the cytoplasm [10,11], indicating particular procedures for their transportation and localization [12]. Thus, ciRNAs and EIciRNA are located in the nucleus, ecircRNAs are abundant in the cytoplasm and exosomes, f-circRNAs have been identified in all cellular compartments, while mecciRNAs are located in the mitochondrial milieu [13]. The discovery of circRNAs in the cytoplasm proposes an action in transcriptional and post-transcriptional control; in fact, some circRNAs regulate gene expression through the modification of pre-mRNA splicing and stability, and the modulation of transcription [14].

**Figure 1:**
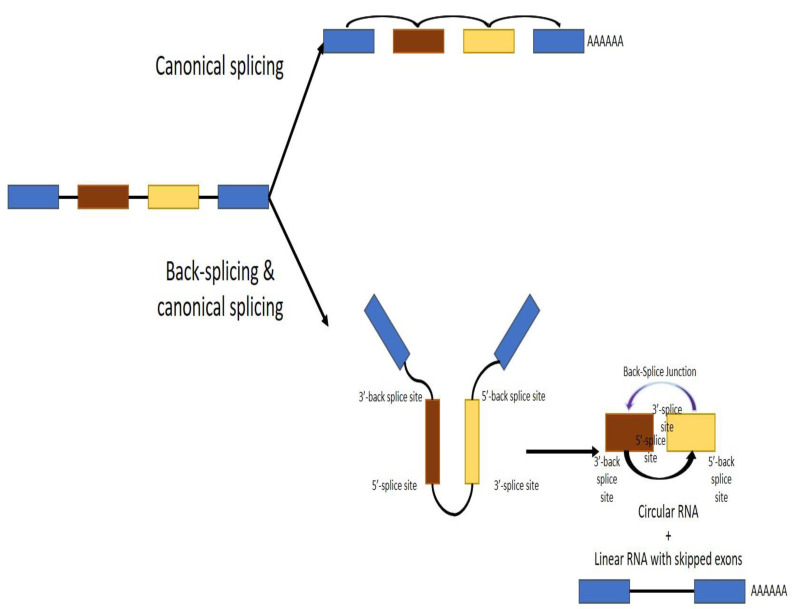
Biogenesis of circular RNAs.

Several studies indicated that circRNAs have a specific genesis, which is different from traditional splicing of linear RNA. In spite of general similarities, emerging data have recognized distinctive characteristics of circRNA formation. CircRNAs are generated by the precursor-mRNA-back-splicing of the exons of genes in eukaryotes. Back-splicing circularization is a further form of alternative splicing. Recent findings have demonstrated that back-splicing needs spliceosomal machinery and that the control of circRNA development is regulated by both cis-regulatory elements and trans-acting factors. The level of steady-state circRNA expression in cells can be influenced by several factors. In fact, regulation of circRNA biogenesis starts from and is coupled with the transcription of circRNA-producing pre-mRNA by Pol II. Moreover, cis- and trans-regulatory elements can further modify the effectiveness of back-splicing, which is catalyzed by spliceosomal machinery. These elements include intronic complementary sequences flanking circle formation exons, core spliceosomal components, and other regulatory RNA-binding proteins. Finally, circRNA turnover has also an effect in their expression levels. Back-splicing of circRNA-forming exons could happen both co- and post-transcriptionally [15,16] (Figure 1).

After being generated, circRNAs tend to establish 16–26 base pair intra-molecularly imperfect RNA duplexes and can be degraded by RNase L [17]. Nevertheless, due to their closed structure, circRNAs are resilient to exonuclease RNase R [18]; thus, compared with linear RNA, circRNAs are extremely stable, and are present in the tissues in concentrations more than 20 times those of their linear parent genes [19,20]. In vitro, the half-life of circRNAs is longer than 48 h, but circRNAs may demonstrate an even longer half-life in vivo [21,22]. However, the processes of circRNA metabolization in vivo are indeterminate, although a recent study stated that the RNA change of N6-adenosine methylation stimulates the engagement of endonucleases to decay circRNAs [23]. It was also reported that the circRNAs are totally metabolized by RNase L upon poly(I:C) stimulation or viral contact [17], but circRNAs could also be discharged from cells via exocytosis, as circRNAs have been identified in exosomes, although it is uncertain if their elimination via exosomes can decrease their intracellular concentrations [24].

**Figure 2:**
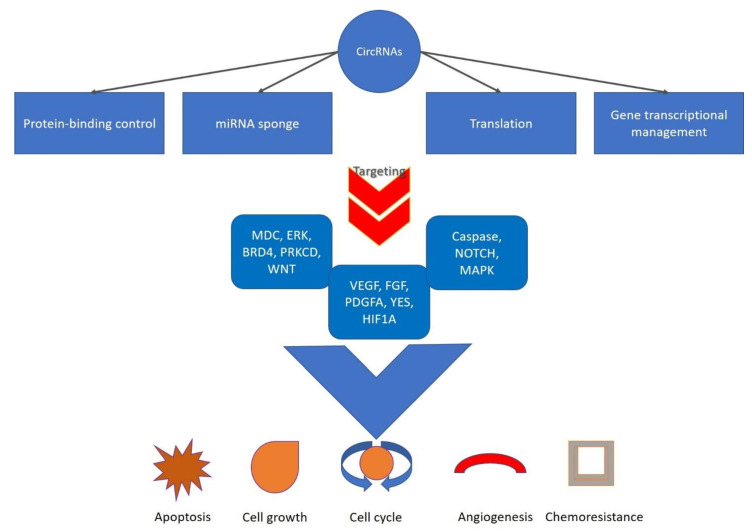
circRNAs carry out diverse biological actions by diverse mechanisms.

As far their functions, several experiments have demonstrated that circRNAs carry out various biological actions by different mechanisms via translation, protein-binding control, sponging of miRNAs, and gene transcriptional management [25] (Figure 2). Among these processes, the most well-studied system is that circRNAs operate as the molecular sponge of miRNA [26]. In particular, circRNAs can competitively join miRNAs and increase the expression of miRNA target genes, thus managing gene expression, or can control RNA-binding-protein function by generating RNA–protein complexes [27,28].

## 2. CircRNAs and Cancer

CircRNAs have cell-, tissue- or disease-context-determined activities, so the same circRNA may have contrasting effects in different circumstances [29]. However, several studies reported that circRNAs were aberrantly expressed in cancer tissues and their alteration was implicated in the occurrence and diffusion of tumors as they have been shown to be able to control cancer dynamics in different malignancies [30].

As far as the causes of circRNA imbalances in neoplastic diseases, there are different modalities to alter the circularization of circRNAs during the cancer process, such as the mutation of trans-acting elements and cis-acting factors, that may alter the expression of circRNAs [31].

CircRNA expression configurations have been evaluated in several solid cancers, including epithelial ovarian cancer, breast cancer, and esophageal squamous cell cancer, and specific circRNAs were revealed to be involved in the genesis of these tumors [32,33,34]. As for hematological malignancies, a general evaluation of circRNA expression patterns revealed 464 altered circRNAs (317 were decreased and 147 were augmented) in acute myeloid leukemia (AML) subjects compared with normal subjects, and among these circRNAs, circ_0004277 was confirmed to be positively correlated with outcomes [35]. Furthermore, circ-CBFB participated in the cell growth while reducing the programmed cell death of chronic lymphocytic leukemia cells via affecting miR-607/FZD3/Wnt/beta-catenin signaling [36].

In this review, we highlight the emergent role and the controlling systems by which circRNAs influence multiple myeloma (MM) genesis. Additionally, we suggest that circRNAs can be considered as potential diagnostic and prognostic markers, can induce chemoresistance, and might represent novel therapeutic targets for multiple myeloma.

## 3. CircRNAs and Multiple Myeloma

Multiple myeloma represents 15% of hematological malignancies with 4.5 to 6 yearly cases per 100,000 subjects, and about 86,000 new MM cases reported yearly [37].

Despite enormous progress in the diagnosis, prognosis, and therapy of MM in recent years [38,39,40,41,42], the disease is still incurable [43]. Moreover, MM does not present clear symptoms in the early phase of disease, and the early detection of the disease is challenging with existing examinations [44]; therefore, it is essential to discover new markers and novel MM-correlated targets through investigating its primary pathogenesis. Therefore, several experiments have established the essential action performed by ncRNAs in the genesis of the disease. For instance, several miRNAs were reported to control MM progression as well as chemoresistance to MM drugs [45,46,47].

Several studies have also demonstrated the prospect of employing circRNAs as helpful diagnostic and prognostic markers in MM, such that a kind of circRNA profile of the MM can be obtained. In fact, a study reported that a circRNA signature was capable of discriminating MM subjects from healthy controls (HCs), and there were 122 increased and 260 decreased circRNAs (in MM subjects compared with HCs), which were involved in altered signaling pathways such as vascular endothelial growth factor (VEGF) and MAPK pathways [48]. Furthermore, circ-PTK2 and circ-RNF217 were related to an inadequate therapeutic response, whereas circ-AFF2 was correlated with a positive treatment response. Several factors could justify these findings as these circRNAs might modify cell sensitivity to chemotherapy and influence chemoresistance via affecting miRNAs. For instance, circ-AFF2 can sponge miRNA-638, once reported to provoke chemoresistance in breast cancer patients, thus reducing drug resistance and improving prognosis in MM subjects [49]. These results were validated by a different report stating that circPTK2 was present in MM cell lines, enhancing MM cell vitality and diffusion and inhibiting programmed cell death. Furthermore, circ-PTK2 controlled miRNA-638 and influenced MM cell activity, stimulating MEK, ERK, and WNT b-catenin signaling pathways [50].

Previous analyses also suggested a modified presence of circ-MYBL2, a circRNA originating from MYBL2, in acute myeloid leukemia and cervical tumors [51,52]. A study assessed circ-MYBL2 in MM patients, and it was remarkably reduced in MM bone marrow and serum compared with healthy controls [53]. Moreover, decreased circ-MYBL2 concentrations were strictly related to advanced clinical stage and poor prognosis, and serum levels were extremely precise in diagnosing MM. Exogenous circ-MYBL2 administration markedly inhibited MM cell survival, DNA production, and proliferation. It was reported that circ-MYBL2 exercised its MM-suppressing action by modifying the amount of phosphorylation of its linear isoform, in which circ-MYBL2 accelerated the joining of cyclin F to MYBL2, inhibiting MYBL2 phosphorylation and stimulation, thus reducing the transcription of several growth-correlated oncogenes. Relevantly, increased circ-MYBL2 decreased the tumor extent of subcutaneous xenografts in experimental animal models.

A different study analyzed another circRNA, circ-CDYL, which was considerably increased in MM tissue and plasma samples and offered great diagnostic and prognostic value [54]. A functional study demonstrated that circ-CDYL enhanced the survival of MM cells and increased DNA synthesis while suppressing programmed cell death. As far as the mechanisms, cytoplasmic circ-CDYL sponged miR-1180 to increase yes-associated protein (YAP) [55,56], thus helping MM progression. miRNA-1180 was reported to be severely reduced in MM and was suppressed by circ-CDYL, and the silencing of miRNA-1180 rescued the reduced aggressive phenotype provoked by circ-CDYL decrease, thus proposing that miRNA-1180 can act as an inhibiting factor in MM. In addition, these findings displayed that YAP, the main effector of the Hippo signaling pathway, was a target gene of miRNA-1180. YAP is often activated in neoplastic diseases, including MM, and an alteration of YAP can stimulate cell proliferation and drug resistance. In the reported study, YAP was decreased in circ-CDYL knockdown cells, and miRNA-1180 silencing reverted this action, suggesting the existence of a regulatory axis of circ-CDYL/miR-1180/YAP in MM cells [54], and the presence and function of this axis was confirmed in vivo by employing a xenograft tumor model. Thus, circ-CDYL is new promoter of MM, and affecting circ-CDYL and its signaling pathway might represent a therapeutic possibility.

Other experiments have explored the effects of different specific circRNAs on the onset and progression of MM. For instance, circ_0007841 was highly expressed in bone marrow (BM) plasma cells of MM subjects and MM cell lines compared with normal controls and normal plasma cell line nPCs. Circ_0007841 stimulated cell growth and inhibited the programmed cell death of MM cells. miRNA-338-3p was a target of circ_0007841 in MM cells and quickened the advancement of MM via miRNA-338-3p. In fact, BRD4 could join miRNA-338-3p in MM cells and this miRNA exerted an anti-MM effect via targeting BRD4, while circ_0007841 increased the stimulation of PI3K/AKT signaling through the miRNA-338-3p/BRD4 axis [57]. Thus, circ_0007841 acted as an oncogene to stimulate the growth and cell cycle and inhibit the programmed cell death of MM cells via segregating miRNA-338-3p to increase the expression of BRD4. These effects were confirmed in other studies and in other neoplastic diseases, including ovarian cancer [58,59].

A different biomarker of MM activity and progression is circ_0000142, which is highly expressed in MM patients, and its high levels was correlated with the advanced International Staging System (ISS) and the Durie–Salmon staging system [60]. Increased concentrations of circ_0000142 enhanced MM cell growth and diffusion and inhibited programmed cell death, while knocking down circ_0000142 restored these effects. As far as the mechanism, circ_0000142 worked as a competitive endogenous RNA, targeting miRNA-610 and controlling AKT3 expression [61].

The human plasmacytoma variant translocation 1 (PVT1) gene codes for both circRNAs and linear ncRNAs. It is involved in different signaling pathways and has relevant effects on several types of cancer. Increased PVT1 concentrations were also found in MM BM cells compared with normal subjects, and mainly in MM patients with MYC mutations. PVT1 knockdown in MM cell lines suppressed cell growth and stimulated programmed cell death [62] via the re-establishment of miRNA-203a expression. In fact, PVT1 operates as an miRNA-203a sponge and inhibition of miRNA-203a restored the PVT1- knockdown phenotype. A similar action was proposed for circPVT1, the ectopic generation of which increased the growth of MM models, inhibited programmed cell death, and enlarged the stem cell compartment [63]. Furthermore, other findings suggest a circPVT1 effect in treatment response [64,65].

Clinical significance might also be found for circ_0000190, a circRNA located in the cytoplasm and decreased in both BM and peripheral blood, while the target of circ_0000190, miRNA-767-5p, was increased, suggesting a negative correlation between them [66]. Circ_0000190 reduced cell survival and growth and provoked an increase in programmed cell death of MM cells [67]. Mitogen-activated protein kinase 4 (MAPK4) is a target of miRNA-767-5p, and increased expression of miRNA-767-5p stimulated cell proliferation by modifying MAPK4. These results were confirmed in vivo in an MM animal experimental model, where dispensation of circ_0000190 reduced tumor proliferation and diffusion. These findings demonstrated that the ability of circ_0000190 to defend against MM was obtained via the inhibition of miRNA-767-5p, which might be a cancer driver via affecting MAPK4. 

A different experiment confirmed these data, and circ_0000190 was negatively correlated with ISS stages, and with several biomarkers, such as beta-2-microglobulin, lactate dehydrogenase, and serum creatinine; the opposite trend was reported for miRNA-767-5p. As far as clinical response, circ_0000190 was correlated with an increased overall response rate (ORR), better overall survival (OS) and progression free survival (PFS), while miRNA-767-5p was correlated with a poor prognosis with reduced complete response (CR) and ORR as well as worse PFS and OS [68]. Thus, circ_0000190 and its target miRNA-767-5p are correlated with risk stratification and prognosis in MM subjects.

A correlation with clinical findings in MM subjects was also assessed for the expression of circ_0001821 in the BM and MM cell lines [69], where its concentrations were increased compared with healthy controls, and its levels were correlated with bone disease, hemoglobin, and Beta-2-microglobulin. In MM subjects aged ≥60 years, increased circ_0001821 demonstrated lower OS compared with MM patients with lower circ_0001821 expression. Moreover, the concentration of caspase-3 protein was lower in MM subjects with high circ_0001821 expression than in those subjects with lower circ_0001821. In fact, an increased expression of circ_0001821 provoked an inhibition of MM-cell programmed cell death, while knockdown of circ_0001821 increased MM-cell apoptosis. Hence, circ_0001821 has an oncogenic effect in MM by controlling cell growth and apoptotic dynamics [69].

Other interesting studies have verified the predictive capability of circRNAs in MM. In previous experiments, MM subjects who presented high C-KIT (CD117) expression were reported to have a better outcome compared with subjects who had low C-KIT expression [70], and 12 circRNAs derived from the C-KIT gene were displayed in K562 cells.

The expression of circ_0069767 was remarkably higher in MM patients than in healthy subjects, but MM subjects with higher expression of circ_0069767 had longer PFS and OS, as if the increased presence of the circRNA caused a reduction of growth and diffusion and increased programmed cell death; moreover, knockdown of circ_0069767 provoked the opposite biological effects. As far as the mechanisms via circ_0069767 exert their actions, it was demonstrated that this circRNA, by sponging miRNA-636 in MM cells, controls cell generation [71].

Finally, the presence of circRNA_101237 in MM cell lines and in the BM of MM patients with recurrent or refractory disease was remarkably increased, especially in patients positive for 1q21 amplification, p53 or 13q14 deletion, and t(4,14) and t(14,16). Furthermore, this circRNA was strictly correlated with the outcomes of MM subjects, as its increased expression was linked with shorter OS and PFS. Bioinformatics evaluations recognized circRNA_101237 networked with 11 miRNAs and 10 candidate mRNAs. This evidence might explain the mechanism of action of this circRNA and its value as a new marker for MM as well as its possible effect in the occurrence and development of MM [72].

The importance of angiogenesis in cancer and hematological malignancies is unquestionable [73,74,75]. However, to date, the practice of antiangiogenic treatments in MM patients has been absolutely inadequate [76]. Some studies have tried to highlight whether circRNAs could act with different mechanisms with respect to their action on proliferation and apoptotic dynamics and have demonstrated that they could also interfere with angiogenic mechanisms.

**Figure 3:**
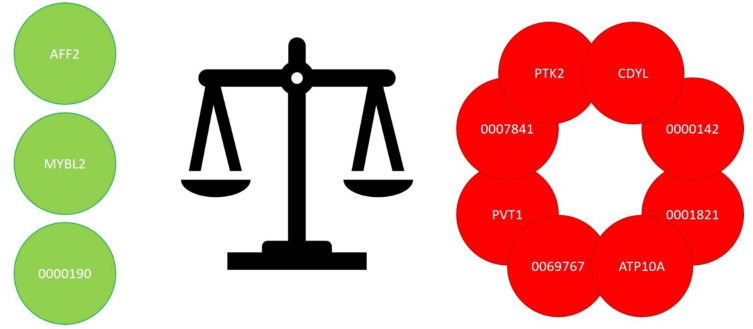
Reduced (green) and increased (red) circRNA in MM.

Exosomal circRNAs were reported to be essential factors for driving angiogenesis in tumors. An experiment recognized a difference in the expression of circRNAs in exosomes from the blood of MM subjects for evaluating prognostic significance. These findings established that the levels of circ-ATP10A were remarkably increased in MM subjects [77]. The bioinformatics assessment indicated that circ-ATP10A can operate as a miRNA sponge and controls the concentrations of several growth factors, such as hypoxia-inducible factor-1alpha (HIF1A), platelet-derived growth factor subunit A (PDGFA), VEGFB, and fibroblast growth factor (FGF), while the circ-ATP10A concentration was correlated with BM microvessel density. These effects were obtained by targeting miRNA-6758-3p/miRNA-3977/miRNA-6804-3p/miRNA-1266-3p/miRNA-3620-3p [77] (Table 1, Figure 3).

### 3.1. CircRNAs and Chemoresistance in MM

In spite of the progress with antimyeloma therapy, the occurrence of chemoresistance is still the main reason for MM relapse [78]. The conditions causing chemoresistance are composite mechanisms, including increased drug efflux pumps efficacy, reduced drug levels, modification in DNA repair, changes in growth and programmed cell death [79], and all types of ncRNAs appear to have a relevant effect on the occurrence of MM drug resistance [80].

The protracted use of BTZ may cause the presence of chemoresistance in MM cells, and to understand the mechanisms of such drug resistance, a study evaluated the correlation between the aforementioned circRNA_101237 and BTZ resistance. In vitro studies demonstrated that this circRNA was increased in BTZ-resistant cell lines and that circRNA_101237 increase was correlated with an inadequate response to BTZ in MM subjects, with a decrease in M protein reduction after therapy [72].

Again, as part of the research performed to examine the mechanisms of drug resistance to BTZ, Wang et al. evaluated the role of circ_0007841 in MM [81], also assessing its correlations with sJAG1, a cell ligand connected with the Notch signaling pathway, which is implicated in MM progression [82]. In MM BM samples, increased concentrations of circ_0007841 and JAG1 and a reduction of miRNA-129-5p were discovered. Circ_0007841 knockdown drastically reduced cell growth, increased programmed cell death in vitro, reduced chemoresistance to BTZ, and decreased tumor progression in vivo. The study provided evidence that circ_0007841 targeted several miRNAs, such as miRNA-129-5p, positively controlled JAG1 production through sponging miRNA-129-5p, and suppression of this miRNA upregulated the effect of the silencing of circ_0007841 on MM cells. Thus, circ_0007841 might be useful as a possible therapeutic target in MM [81].

CircRNA itchy E3 ubiquitin protein ligase (circITCH) is a circRNA having a relevant effect in the occurrence of several tumors [83], and this circRNA was reduced in MM BB samples and cell lines as well as in BTZ-resistant MM cells and MM patients with poor prognosis. Increased concentrations of circITCH enhanced the sensitivity of BTZ-resistant MM cells to BTZ in both in vitro and in vivo studies. Moreover, circITCH was recognized as a sponge for miRNA-615-3p, and PRKCD as a direct target of miRNA-615-3p. CircITCH might operate via a miRNA-615-3p/PRKCD axis, presenting a new possible system for preventing BTZ resistance in MM subjects [84].

Finally, Liu et al. evaluated the possible effect of circular RNA chaperonin enclosing TCP1 subunit 3 (circ-CCT3) in BTX resistance [85]. Circ-CCT3 and BRD4 were increased, while miRNA-223-3p was reduced in BTZ-resistant MM subjects and cells. The silencing of circ-CCT3 enhanced the sensitivity of cells to BTZ by changing the expression of miRNA-223-3p, which fostered BTZ sensitivity by suppressing BRD4.

Among the novel drugs used in the treatment of MM, immunomodulator drugs (IMiDs) have also dramatically enhanced the survival of MM subjects, and drug resistance to IMiDs represents the main problem in the therapy of these patients [86].

A report described genome expression configurations of circRNAs in IMiD-sensitive and IMiD-resistant MM cells [87]. The authors found that genome circRNA expression revealed IMiD sensitivity and that ciRS-7 was the most decreased circRNA in patients with acquired resistance. The reduction of ciRS-7 connected with increased methylation concentrations of the promoter CpG island of its host gene, LINC00632, and administration of an EZH2 inhibitor (EPZ-6438) and a DNA methyl transferase inhibitor (5-azacytidine) re-established the production of LINC00632 and ciRS-7, which also restored the IMiD sensitivity of the cells.

Other studies have clarified the presence of correlations between some circRNAs and other drugs used in the treatment of MM, such as circ_0007841, which was reported to enhance doxorubicin resistance in MM cells via increasing ATP-binding cassette transporter G2 (ABCG2) expression [88]. Its expression is increased in doxorubicin-resistant cells with respect to parent cells, and the silencing of circ_0007841 in resistant cells could decrease the half-maximal inhibitory dose, suggesting a decrease in drug resistance. These findings propose that the combined use of an ABCG2 inhibitor and a circ_00078416 inhibitor could be a possible treatment for MM cells [88] (Table 2).

Finally, steroids are a cornerstone of MM therapy, and circPVT1 was increased in glucocorticoid-resistant cells, while its reduction increased sensitivity to glucocorticoid administration, stimulated programmed cell death, and blocked cell growth in resistant cell lines and xenograft models via an increase in caspase-3 and PARP and a decrease in BCL2 [63].

### 3.2. CircRNA and MM Complications

During MM disease, light chains and polysaccharide compounds are accumulated in tissues and can harm organ activities, causing kidney failure, anemia, hypercalcemia, lithic lesions, and cardiac alterations [89], while the same anti-MM drugs can provoke the occurrence of organ damage such as peripheral neuropathy and heart disease.

Peripheral neuropathy (PN) is a complication of MM, which negatively influences MM patients’ quality of life. Several analyses have reported that about 20% of MM subjects present with PN at the onset of their disease, and almost 75% experience chemotherapy-induced PN (CIPN).

Exosomes are small extracellular vesicles with a size between 30 and 100 nm and can be carried on circRNAs, mRNAs, and other noncoding RNAs [90,91] and are transferred via endocytosis or direct union with the target cell membrane, thus allowing intercellular interactions between the cell and remote cells or far tissues [92]. This condition is generally recognized as a relevant promoter of cancer progression as exosomes can provoke the stimulation, growth, and apoptosis of target cells [92]. Various studies described exosomes as also being implicated in MM tumorigenesis [93].

An experiment reported that the levels of serum exosomal (exo) circMYC, a circRNA originating from the MYC gene, were remarkably increased in MM patients compared with normal controls, while the level of circMYC in circulating exosomes in BTZ-resistant subjects was greater than that in non-resistant subjects [94]. Moreover, the amount of exo-circMYC was associated with the Durie–Salmon and the ISS, and with deletion 17p, and t(4;14). Statistical analysis demonstrated that a high exo-circMYC concentration was an independent predictor of poor outcomes in MM subjects, with greater relapse rates, greater mortality percentages, and reduced OS and PFS compared with patients with low exo-circMYC expression [94]. 

Zhang et al. evaluated the relationship between serum exo-circRNAs and MM-related PN [95] and found 265 increased circRNAs and 787 regulated circRNAs with at least a two-fold modification in their expression in MM subjects compared with normal subjects. Bioinformatics examination suggested that increased circRNAs possibly accelerated MM-related PN. Furthermore, analysis revealed that chr2:2744228-2,744,407+ might provoke MM-derived PN through the downstream miRNA and the ionotropic glutamate receptor GRIN2B axis. Increased chr2:2744228-2,744,407+ in the serum exosomes of MM subjects might cause a decrease in miRNA-6829-3p, an increase in GRIN2B in the serum, and also suppressed cell survival. Furthermore, a correlation evaluation showed that the level of chr 2:2744228-2,744,407+ was positively associated with the occurrence and clinical findings of PN, suggesting that exo-circRNA might represent a possible new therapeutic target for MM-related PN [95].

Furthermore, clinical reports showed that up to 50% of MM subjects present with heart damage, and cardiac complications represent one of the most severe problems in MM subjects and can lead to heart failure provoked by cardiac amyloidosis, anemia, or by the direct or indirect effects of some drugs employed in MM treatment [96,97].

A study evaluated the relationships between exo-circRNAs and MM-related heart impairment [98]. Bioinformatics assessment demonstrated that enhanced expression of circRNAs were capable of inducing MM-related myocardial failure. Exo-circ-G042080 was significantly expressed in the blood of MM patients and its expression was positively associated with MM-correlated myocardial damage. The negative effect of circ-G042080 might be due to a downstream miRNA/TLR4 axis. In vitro studies demonstrated that this axis might be shown in H9C2 cells cultured with exosomes and it is able to induce aberrant autophagy. Therefore, exo-circRNAs might represent a novel diagnostic biomarker of MM-related heart damage and a possible therapeutic target [98].

## 4. Conclusions

New, non-invasive diagnostic markers for MM diagnosis are particularly relevant as the prompt identification of MM is key to enhancing survival. As such, several studies indicated that the circulating transcriptome is a precious spring of such markers [99,100]. Moreover, due to their great stability, circRNAs are better indicators of disease with respect to their linear RNA as diagnostic and prognostic markers and as possible regulators of chemoresistance in MM cells.

However, in spite of the fact that huge progress has been made in the study of circRNAs, there are several aspects of circRNAs that need to be investigated before they can be incorporated into clinical practice, and numerous challenges remain to be addressed. Indeed, although different roles of circRNAs have been described, the intimate mechanisms of these actions in MM require further analysis. Furthermore, owing to the huge amount of circRNAs, checking significant circRNAs is a long procedure and the low level of circRNAs in biological samples and imperfect identification techniques prevent their extensive clinical use [101]; moreover, procedural issues such as cross-hybridization questions in microarrays and PCR amplification bias can hinder results [102,103].

Additionally, even the transfer of the findings obtained in in vitro experimental animal models to in vivo models appears troublesome as low evolutionary preservation [104] reduces the chances to employ animal models to analyze their function. Still, when conducting in vitro loss-of-function analyses employing procedures such as RNA interference (RNAi), several problems occur as the nuclear localization of most circRNAs makes RNAi less successful [105,106]. An effective instrument to generate stable knockouts is the clustered regularly interspaced palindromic repeats (CRISPR) technique [107]. However, investigators must be conservative when employing this procedure for knockdown of circRNAs as it is hard to prevent changes in the expression of protein-coding genes from the same locus [108].

However, despite the limitations mentioned above, there is no uncertainty that the analysis of circRNAs opens novel possibilities in the understanding of the pathophysiology of MM and in the ability to predict response to therapy, and that it also provides new therapeutic targets that could guarantee better survival for patients with MM.

## Figures and Tables

**Table 1:** Effects of circRNA in multiple myeloma.

CircRNA	Expression in MM	Target	Mechanism	Ref.
circ-AFF2	Reduced in poor prognosis MM patients	miRNA-638	Effect on sensitivity to chemotherapy	[49]
circ-PTK2	Augmented	miRNa-638	MEK, ERK and WNT b-catenin signaling pathways	[50]
circ-MYBL2	Reduced	Joining of cyclin F to MYBL2	Inhibition of MYBL2 phosphorylation	[53]
circ-CDYL	Augmented	miR-1180	Changed yes-associated protein	[54]
circ_0007841	Augmented	miRNA-338-3p	Augmented expression of BRD4	[57]
circ_0000142	Augmented	miRNA-610	AKT3 expression	[61]
circPVT1	Augmented	miRNA-203a	Apoptosis	[63]
circ_0000190	Reduced	miRNA-767-5p	Mitogen-activated protein kinase 4	[67,68]
circ_0001821	Augmented		Caspase-3 protein	[69]
circ_0069767	Augmented	miRNA-636	Apoptosis	[71]
circ-ATP10A	Augmented	miRNA-6758-3p, miRNA-3977, miRNA-6804-3p, miRNA-1266-3p, and miRNA-3620-3p	Angiogenesis. Effects on hypoxia-inducible factor-1 alpha, platelet-derived growth factor subunit A, vascular endothelia growth factor B, and fibroblast growth factor	[77]

**Table 2:** CircRNAs and chemoresistance in multiple myeloma.

circRNA	Drug	Mechanism	Target	Ref.
circRNA_101237	BTZ			[72]
circ_0007841	BTZ	sJAG1, notch signaling pathway	miRNA-129-5p	[81]
circITCH	BTZ	PRKCD	miRNA-615-3p	[84]
circ-CCT3	BTZ	BRD4	miRNA-223-3p	[86]
ciRS-7	IMiDs		methylation of the promoter CpG island of LINC00632	[87]
circ_0007841	Doxorubicin	ATP-binding cassette transporter G2		[88]
circPVT1	Glucocorticoids	Apoptosis	Caspase-3 and PARP, BCL2	[63]

## Abbreviations

ABCG2	ATP-binding cassette transporter G2
BM	bone marrow
BTZ	bortezomib
CIPN	chemotherapy-induced PN CIPN
circ-CCT3	circular RNA chaperonin-inclosing TCP1 subunit 3
circITCH	circRNA itchy E3 ubiquitin protein ligase
circRNA	circular RNA
ciRNAs	circular intronic RNAs
CR	complete response
CRISPR	clustered regularly interspaced palindromic repeats
ecircRNAs	exonic circRNAs
EIciRNAs	exon-intron circRNAs
f-circRNAs	fusion circRNA
FGF	fibroblast growth factor
HCs	healthy controls
HIF1A	hypoxia-inducible factor-1alpha
IMiDs	immunomodulator drugs
LncRNAs	long ncRNAs
mecciRNAs	mitochondria-encoded circRNA
MM	multiple myeloma
ncRNAs	non-coding RNAs
OS	overall survival
PDGFA	platelet-derived growth factor subunit A
PFS	progression free survival
PN	peripheral neuropathy
PVT1 Human	plasmacytoma variant translocation 1
rt-circRNAs	read-through circRNAs
VEGF	vascular endothelial growth factor
YAP	yes-associated protein
